# Combined Effects of Dietary Soybean Lecithin With Vitamin C or Inositol on Growth, Gonadal Development, and Intestinal Microbiota in Longsnout Catfish

**DOI:** 10.1155/anu/4061190

**Published:** 2026-09-22

**Authors:** Ming-Hua Xie, Yu-Lin Zhou, Min Liu, Zhi Li, Peng Zhen, Jun Tian, Yi Gong, Xiao-Juan Zhang, Dong Han, Li Zhou, Jian-Fang Gui, Zhong-Wei Wang

**Affiliations:** ^1^ State Key Laboratory of Breeding Biotechnology and Sustainable Aquaculture, Institute of Hydrobiology, Chinese Academy of Sciences, Wuhan 430072, China, cas.cn; ^2^ College of Advanced Agricultural Sciences, University of Chinese Academy of Sciences, Beijing 100049, China, ucas.ac.cn

**Keywords:** dietary additives, gonadal development, *Leiocassis longirostris*, microbial community structure, transcriptome

## Abstract

To investigate the combined effects of soybean lecithin (SL) with vitamin C (VC) or inositol (INO) on growth, gonadal development, and intestinal microbiota, 2‐year‐old longsnout catfish (*Leiocassis longirostris*) were fed four isonitrogenous and isolipidic diets for 12 weeks, including a control diet, a 4% SL diet, a 4% SL + 0.5% VC (SC) diet, and a 4% SL + 0.5% INO (SI) diet. A two‐way analysis of variance (ANOVA) revealed a significant diet × sex interaction for weight gain rate (WGR) and specific growth rate (SGR) (*p* < 0.05). In females, the SI group exhibited the highest WGR and SGR, while both the SC and SI groups showed significantly higher WGR and SGR than both the SL and control groups in males (*p* < 0.05). All three supplemented groups promoted gonadal development, as reflected by higher gonadosomatic index (GSI) and elevated serum 17β‐estradiol (E2) and progesterone (P4) levels. Both SC and SI groups showed significantly increased total antioxidant capacity (T‐AOC), superoxide dismutase (SOD), catalase (CAT), and alkaline phosphatase (AKP) activities and significantly decreased malondialdehyde (MDA) and cortisol levels compared with the control group (*p* < 0.05). Dietary SC enriched potentially beneficial *Bacteroides* in both sexes. SI enriched potentially beneficial *Cetobacterium* in males. Furthermore, all three supplemented diets reduced the potentially pathogenic *Plesiomonas* in females. Gonadal transcriptome analysis suggested that SL combined with VC or INO may modulate pathways such as the peroxisome proliferator‐activated receptor (PPAR) signaling pathway, biosynthesis of unsaturated fatty acids, estrogen signaling, and histidine metabolism, implying potential roles in gonadal lipid metabolism, chaperone‐mediated protection, and immune homeostasis. Overall, SI was most effective in promoting growth in females, whereas both SC and SI were more effective in promoting gonadal maturation, enhancing antioxidant capacity, and improving intestinal microbiota composition. This study provides theoretical guidance for the development of specialized compound feeds during the gonadal development period in longsnout catfish aquaculture.

## 1. Introduction

Aquaculture is widely recognized as the most efficient food‐producing industry and has abundant species diversification [1–3]. The longsnout catfish (*Leiocassis longirostris*) is a representative species of the genus *Leiocassis* within the family Bagridae. It is widely distributed in major river systems across China, such as the Yangtze River, the Liaohe River, and the Huaihe River [4, 5]. Owing to its nutrient‐rich composition, delicate texture, and pleasant flavor, it has been favored by the Chinese consumers since ancient times [6]. With breakthroughs in artificial breeding techniques and the development of intensive farming models, both the scale and production of its aquaculture have expanded rapidly across East Asia. However, with the further development of the aquaculture industry, how to consistently obtain high‐quality mature broodstock to meet the demand for superior seedlings in large‐scale production has emerged as a critical issue. Gonadal development and maturation typically require substantial accumulation of nutrients; therefore, an adequate and balanced nutritional supply is a key factor in ensuring normal gonadal development in broodstock [7]. Precisely regulating sexual maturation through nutritional approaches represents an economically viable and effective strategy. Consequently, it is crucial to investigate the nutritional status of longsnout catfish during the gonadal development period.

Before entering the reproductive stage, aquatic animals require a substantial accumulation of nutrients, particularly lipids [8]. Lipid nutrients not only provide energy for broodstock and serve as the material basis for ovarian synthesis but also act as precursors for key hormones, regulating the secretion of related sex hormones [9, 10]. Phospholipids, which are present in all lipid classes as critical components of lipoproteins, are essential for maintaining cell membrane integrity and stability and for the proper functioning of the reproductive system [11]. Soybean lecithin (SL), due to its high phospholipid content, accessibility, and low cost, has become an outstanding representative among phospholipids [12]. Studies on striped catfish (*Pangasianodon hypophthalmus*) [9] and red‐claw crayfish (*Cherax quadricarinatus*) [13] have demonstrated that dietary SL supplementation promotes gonadal development and sexual maturation by enhancing sex hormone secretion and facilitating lipid accumulation in the liver and ovaries. However, excessive lipid accumulation in the liver can also easily trigger various inflammatory responses, oxidative stress, and metabolic disorders [14].

Vitamin C (VC), a biological antioxidant involved in hydroxylase reactions, plays a significant role in enhancing cellular resistance to oxidative stress and protecting cytoplasmic components or tissues from oxidative damage. Studies on red sea bream (*Pagrus major*) [15] and milkfish (*Chanos chanos*) [16] have shown that dietary VC supplementation effectively improves growth performance, immune response, and spawning quality. More importantly, dietary VC can also help maintain hepatic lipid homeostasis by reducing excessive lipid accumulation in the liver, thereby supporting normal hepatic function [17, 18]. Inositol (INO), a vitamin‐like functional nutrient, is widely used in diets for various aquaculture species [19, 20]. Metabolites of INO, such as INO triphosphate (IP3), can also act as second messengers involved in regulating ovarian development in animals [21, 22]. Meanwhile, studies on hybrid grouper (♀ *Epinephelus fuscoguttatus* × ♂ *E. lanceolatus*) [14] and Nile tilapia (*Oreochromis niloticus*) [23] have shown that dietary INO supplementation not only effectively improves growth performance and antioxidant capacity but also promotes lipid utilization and reduces hepatic lipid accumulation by modulating lipid metabolism. The established roles of VC and INO in improving growth and regulating lipid metabolism suggest broader physiological significance; however, their involvement in reproductive regulation and their capacity to enhance SL utilization warrant further investigation.

However, current research on the effects of VC and INO on gonadal development in aquatic animals remains limited and lacks depth, and the molecular mechanisms through which SL promotes sexual maturation in fish are still unclear. More importantly, the efficacy of VC and INO in assisting and improving the utilization of SL has not been well‐defined. Therefore, this study integrates intestinal microbiota analysis and gonadal transcriptome sequencing technology to investigate the effects of SL, SL combined with VC, and SL combined with INO on growth, antioxidant capacity, and gonadal development in both female and male longsnout catfish. These findings are expected to provide theoretical guidance for the development of specialized diets during the gonadal development period and contribute to the sustainable development of the aquaculture industry in longsnout catfish.

## 2. Materials and Methods

### 2.1. Experimental Fish

Two‐year‐old longsnout catfish individuals used for artificial propagation were obtained from the National Aquatic Biological Resource Center (Wuhan, China). All experiments and animal treatments were approved by the Institutional Animal Care and Use Committee of the Institute of Hydrobiology (IHB2024‐0405).

### 2.2. Feed Preparation

In the present study, four groups of compound feeds with isonitrogenous (42.0%) and isolipidic (10.4%) content were produced and used as experimental diets for fish (Table 1). Three experimental diets were formulated by supplementing the control diet with 4% SL (SL group), 4% SL + 0.5% VC (SC group), and 4% SL + 0.5% INO (SI group). First, primary feed components (fish meal, soybean meal, cottonseed meal, rapeseed meal, and wheat flour) were ground and then screened through a 40‐mesh sieve. The ingredients were accurately weighed according to the experimental feed formulation. The main components were thoroughly mixed with the trace components, followed by further blending with the weighed fish oil, soybean oil, and SL. The feed ingredients were mixed uniformly in the mixer and stirred continuously while gradually adding water. The uniformly mixed ingredients were processed into 4 mm diameter feed pellets using a granulating machine (SLR‐45, Fishery Machinery and Instrument Research Institute, Chinese Academy of Fishery Sciences, Shanghai, China). The feed pellets were then dried and stored in a −20°C freezer. The isonitrogenous and isolipidic levels and the dosages of 4% SL, 0.5% VC, and 0.5% INO were selected based on previous studies on siluriform fish and other fish species [24–28]. SL (phospholipid content ≥ 95%, Henan Xinbaiweijia Food Technology Co., Ltd.), VC (L‐ascorbyl‐2‐polyphosphate, VC equivalent ≥ 35%, Guangdong Liankun Group Co., Ltd.), and INO (purity ≥ 97%, Guangdong Liankun Group Co., Ltd.) were used as dietary supplements. The proximate composition of the experimental diets was determined following the guidelines of the AOAC (2005). The moisture content was measured by drying samples to constant weight at 105°C (method 930.15). Crude protein was determined using the Kjeldahl procedure (method 984.13) and crude lipid was determined using the Soxhlet extraction technique (method 920.39). The crude fiber was analyzed using an automatic fiber analyzer (A2000i, ANKOM Technology Corp., USA). The content was determined by incineration in a muffle furnace at 550°C for 12 h (method 942.05). The nitrogen‐free extract (NFE) was calculated by difference as follows: NFE = 100 − (moisture + crude protein + crude lipid + crude fiber + ash). Gross energy was measured using an adiabatic oxygen bomb calorimeter (Parr 6200, Parr Instrument Co., Moline, IL, USA) according to the AOAC Method 983.11.

**Table 1:** The formula and proximate composition of the diets (%, dry matter).

Ingredients	Groups			
	Control	SL	SC	SI
Brown fish meal	40.00	40.00	40.00	40.00
Wheat flour	12.00	12.00	12.00	12.00
Soybean meal	10.00	10.00	10.00	10.00
Rapeseed meal	10.00	10.00	10.00	10.00
Cottonseed protein	10.00	10.00	10.00	10.00
Fish oil	3.50	1.50	1.50	1.50
Soybean oil	3.50	1.50	1.50	1.50
Vitamin premixes^a^	0.39	0.39	0.39	0.39
Mineral premixes^b^	5.00	5.00	5.00	5.00
Choline chloride	0.11	0.11	0.11	0.11
Ca(H_2_PO_4_)_2_	2.00	2.00	2.00	2.00
Ethoxy quinoline	0.10	0.10	0.10	0.10
Carboxymethyl cellulose	3.40	3.40	2.90	2.90
Soybean lecithin	0.00	4.00	4.00	4.00
Vitamin C	0.00	0.00	0.50	0.00
Inositol	0.00	0.00	0.00	0.50
Proximate composition				
Crude protein	43.60	44.01	43.79	43.69
Crude lipid	10.27	9.84	10.26	9.46
Ash	12.37	13.01	13.20	13.12
Moisture	9.57	8.19	6.99	7.95
Nitrogen‐free extract	20.07	21.46	23.34	23.21
Gross energy (MJ/kg)	20.02	19.55	19.27	19.58

^a^Vitamin premixes (mg/kg diet): Vitamin B_1_, 20.00; Vitamin B_2_, 20.00; Vitamin B_6_, 20.00; Vitamin B_12_, 0.02; folic acid, 5.00; calcium pantothenate, 50.00; niacin, 100.00; biotin, 0.10; Vitamin A, 11.00; Vitamin D, 2.00; Vitamin E, 100.00; Vitamin K, 10.00; corn starch, 3561.88.

^b^Mineral premixes (mg/kg diet): NaCl, 500.0; MgSO_4_ · 7H_2_O, 8155.6; NaH_2_PO_4_ · 2H_2_O, 12500.0; KH_2_PO_4_, 16000.0; CaHPO_4_·2H_2_O, 7650.6; FeSO_4_ · 7H_2_O, 2286.2; C_6_H_10_CaO_6_·5H_2_O, 1750.0; ZnSO_4_ · 7H_2_O, 178.00 MnSO_4_ · H_2_O, 61.4; CuSO_4_ · 5H_2_O, 15.5; CoSO_4_·7H_2_O, 0.91; KI, 1.5; Na_2_SeO_3_, 0.60; Corn starch, 899.69.

### 2.3. Feeding Management

The feeding trial was conducted at the Liangzi Lake Modern Ecological Fisheries Research Base (Wuhan, China). Prior to the formal feeding trial, a 2‐week acclimatization period was conducted during which the control group diet was administered. Following the adaptation period, the genetic sex of each fish was verified by PCR amplification using the sex‐specific molecular marker for longsnout catfish (Figure S1) [29]. Subsequently, a 12‐week feeding trial was conducted. A total of 120 apparently healthy fish of uniform size, comprising 60 females (345.27 ± 5.79 g) and 60 males (417.48 ± 5.36 g), were selected and randomly distributed into 12 circular fiberglass tanks (~4 m^3^ each; diameter: 1 m and height: 1.3 m). Each treatment group was assigned 3 replicate tanks, with each tank stocked with 5 female and 5 male fish. Throughout the experiment, feeding occurred twice daily at 7:30 and 18:30 until satiation. Water temperature was maintained at 24–30°C, pH at 7.0–7.4, dissolved oxygen > 6 mg/L, and total ammonia nitrogen < 0.5 mg/L.

### 2.4. Sample Collection and Chemical Analyses

Following a 12‐week feeding trial, fish were fasted for 24 h. All individuals were anesthetized with MS–222 (Sigma, USA) before sampling. Body length and final body weight (FBW) were recorded for all fish. From each tank, three females and three males were randomly selected (9 females and 9 males per dietary treatment, with three replicate tanks per treatment) for blood and tissue collection. Blood was drawn from the caudal vein, and the serum was stored at −80°C for later analysis of hormone levels and enzyme activities. These fish were then dissected to weigh the liver, viscera, and gonads for the calculation of hepatosomatic index (HSI), viscerosomatic index (VSI), and gonadosomatic index (GSI). Their gonads were dissected, and tissue sections from the same region were fixed in 4% paraformaldehyde for histological analysis.

The remaining fish in each tank were used for molecular and intestinal microbiota analyses. Ovaries from females and testes from males were dissected for RNA‐seq and reverse transcription quantitative real‐time PCR (RT‐qPCR) analysis. A small portion of the midgut was also collected from these fish for intestinal microbiota analysis. For each tank, gonad tissues and midgut tissues from fish of the same sex were each pooled as one biological replicate, yielding three independent biological replicates per sex per treatment group (*n* = 3), each derived from a separate tank. All tissue samples were immediately placed in RNase‐free tubes and stored at −80°C.

### 2.5. Histological Analysis

Gonadal tissues were fixed in 4% paraformaldehyde for 24 h, dehydrated through a graded ethanol series, embedded in paraffin, sectioned, and stained with hematoxylin and eosin (HE). Using K‐Viewer software (v.1.5.3.1), ovarian and testicular sections from three females and three males per tank in each treatment group were imaged. For ovarian sections, three fields were randomly selected at 100× magnification to count the number of oocytes at different developmental stages and to measure the oocyte diameter. For testicular sections, three fields were randomly selected at 400× magnification to count the numbers of spermatogonia, spermatocytes, secondary spermatocytes, and spermatozoa and to estimate sperm density. ImageJ software was used for quantification.

### 2.6. Sex Hormones and Immune Indicators Analysis

Serum levels of 17β‐estradiol (E2, LB69675B), progesterone (P4, LB010706B), testosterone (T, LB3826B), and androstenedione (A4, LB6308B) were measured using enzyme‐linked immunosorbent assay (ELISA) kits. Alkaline phosphatase (AKP, ADS‐W‐ZM003‐96), superoxide dismutase (SOD, ADS‐W‐KY011) activity, catalase (CAT, ADS‐W‐KY002), total antioxidant capacity (T‐AOC, ADS‐W‐KY005‐96), and malondialdehyde (MDA, ADS‐W‐YH002) were determined using commercial biochemical kits. Cortisol (LB3785B) was detected using an ELISA kit. All kits were sourced from Meimian Biotechnology Co., Ltd. (Jiangsu, China). The experimental procedures were strictly conducted in accordance with the manufacturer’s instructions.

### 2.7. Intestinal Microbiome Analysis

Genomic DNA was isolated from samples using the MagPure Soil DNA LQ Kit (Magan, Guangdong, China) according to the manufacturer’s instructions. The quality and quantity of the extracted DNA were evaluated by agarose gel electrophoresis and a NanoDrop 2000 spectrophotometer (Thermo, USA). The DNA was then used as a template to amplify bacterial 16S rRNA genes via PCR with barcoded primers. The V4–V5 regions of the 16S rRNA gene were amplified using primers 515F (5^′^–GTGCCAGCMGCCGCGG–3^′^) and 907R (5^′^–CCGTCAATTCMTTTRAGTTT–3^′^). PCR products were purified, and libraries were prepared following standard Illumina protocols. Sequencing was performed on an Illumina NovaSeq 6000 platform, generating 250 bp paired‐end reads. After adapter trimming with Cutadapt, reads were processed using the QIIME2 (v.2020.11) pipeline (DADA2) for quality filtering, denoising, merging, and chimera removal. Amplicon sequence variants (ASVs) were obtained, and taxonomic assignment was performed against the Silva database (v.138). Alpha diversity indices were calculated with QIIME2, and statistical comparisons between groups were conducted using R software (v. 3.2.0). Beta diversity was visualized using principal coordinate analysis (PCoA) based on Bray–Curtis distances. One‐way analysis of variance (ANOVA) was first performed to evaluate the overall differences among groups. Raw *p*‐values across all taxa were then adjusted using the Benjamini–Hochberg false discovery rate (FDR) method. Taxa with an FDR *q*‐value < 0.05 were retained for subsequent pairwise comparisons using Tukey’s HSD post‐hoc test, with adjusted *p* < 0.05 considered significant. Library preparation and sequencing were completed by OE Biotech Co., Ltd. (Shanghai, China).

### 2.8. Transcriptome Sequencing and Analysis

Total RNA was extracted from ovary tissues of female fish and testis tissues of male fish using the TRIzol reagent (Invitrogen, USA) following the manufacturer’s instructions. RNA purity and concentration were assessed using a NanoDrop 2000 spectrophotometer, and RNA integrity was evaluated with an Agilent 2100 Bioanalyzer. Sequencing libraries were constructed using the VAHTS Universal V10 RNA‐seq Library Prep Kit (Vazyme, China). Paired‐end sequencing (150 bp) was performed on an Illumina NovaSeq 6000 platform. Raw reads in the FASTQ format were first processed using fastp to remove low‐quality reads and obtain clean reads. The clean reads were then mapped to the reference genome using HISAT2. Raw read counts for each gene were obtained using HTSeq‐count, and FPKM values were calculated for expression visualization. Differential expression analysis was performed using DESeq2 based on the raw counts. The thresholds for differentially expressed genes (DEGs) were set at *q*‐value < 0.05 and |log_2_ fold change| > 1 (fold change > 2 or <0.5). Hierarchical cluster analysis of DEGs was performed using R (v3.2.0) to illustrate gene expression patterns across groups. GO and KEGG pathway enrichment analyses were carried out based on the hypergeometric distribution, and significantly enriched terms were visualized as bar or bubble charts. The transcriptome sequencing and data analysis were completed by OE Biotech Co., Ltd. (Shanghai, China).

### 2.9. Validation of DEGs by RT‐qPCR

Total RNA was extracted from ovary tissues of female fish and testis tissues of male fish using the TRIzol reagent, followed by reverse transcription to synthesize cDNA. qPCR was performed with iTaq Universal SYBR Green Supermix (Bio‐Rad) on a CFX96 Real‐Time PCR System (Bio‐Rad). The 20 μL reaction mixture contained 1 μL cDNA, 10 μL iTaq Universal SYBR Green Supermix, 0.5 μL forward primer (10 μM), 0.5 μL reverse primer (10 μM), and 8 μL ddH_2_O. All samples were run in triplicate. The thermal cycling conditions were as follows: initial denaturation at 95°C for 30 s; 40 cycles of 95°C for 5 s and 59°C for 30 s; melting curve analysis from 65 to 95°C, with fluorescence signal collected continuously at a ramp rate of 0.5°C per 5 s. Three biological replicates per sex per treatment group were used. Each sample was run in triplicate. Relative gene expression levels were calculated using the Livak (2^−ΔΔCt^) method with *β-actin* as the internal control. Primer sequences and annealing temperatures for all analyzed genes are listed in Table 2. Pearson correlation analysis was performed to assess the consistency between RT‐qPCR and RNA‐seq.

**Table 2:** RT‐qPCR primer sequences.

Gene name	Primer sequences (5^′^–3^′^)		Annealing temperature (°C)	Slope	Efficiency (%)
Actin, beta 1 (*β*‐actin)	F	CCTGACTCTGAAGTACCCCATTGAGC	60	−3.30	100.98
	R	CAGAAGCGTAAAGGGACAGCACGG			
Fatty acid desaturase 2 (*fads2*)	F	ACCACGATAAACACGACGATTGGCTC	61	−3.24	103.52
	R	CCATACTTCTCACAGAGTTCTCGTACTTG			
Stearoyl‐CoA desaturase 1 (*scd1*)	F	CTCAGGTACACACTTGTTCTAAATGCCAC	61	−3.19	105.88
	R	AGTGGGGACACGACGGCAGTC			
Fatty acid transport‐related genes solute carrier family 27 member 2 (*slc27a2*)	F	GCGGTGGAGGAAGTACTTCCTGTCT	61	−3.50	93.10
	R	CACAGCTGCTTTAGGAAGACCAGTG			
Heat shock protein 70 (*hsp70*)	F	GCTCATCAAACGCAACACCACCATC	61	−3.26	102.78
	R	CCTGTGCTTTTGTCCACGGCAGA			
Heat shock protein family A member 8 (*hspa8*)	F	GAGGATGATGTTCAGCGTGACAAGGT	61	−3.24	103.42
	R	CATTCCCCCTGGCATGCCTCCA			
Histamine *N*‐methyltransferase (*hnmt*)	F	GAATCCCTTACGAGAGCCATGTTTTTGAG	60	−3.35	98.89
	R	GTCAATCACTAAGGCCTCCATGTTGC			

### 2.10. Calculations and Statistical Analysis

Based on the recorded data, survival rate and growth performance indicators, including weight gain rate (WGR) and specific growth rate (SGR), as well as GSI, HSI, VSI, and CF were calculated. The calculation formulas are as follows:

WGR=Final body weight−Initial body weightInitial body weight×100,

SGR=lnFinal body weight−lnInitial body weightdays×100,

GSI=Gonadal weightFinal body weight×100,

HSI=Liver weightFinal body weight×100,

VSI=Visceral weightFinal body weight×100,

CF=Final body weightBody length3×100,

Survival rate=Final number of fishInitial number of fish×100.

Quantitative gonadal histological parameters and sex hormone levels were compared among dietary groups separately for each sex using one‐way ANOVA. All other variables were analyzed by two‐way ANOVA with diet and sex as fixed factors, using a tank as the experimental unit. When a significant diet × sex interaction was detected, two separate one‐way ANOVAs were performed to compare dietary groups within each sex, with post‐hoc comparisons conducted using Tukey’s HSD test. In addition, independent‐sample *t*‐tests were performed to compare females and males within each dietary group. When no significant diet × sex interaction was detected, the main effects of diet and sex were examined on the pooled data, with Tukey’s HSD test used for diet comparisons. Data are presented as the mean ± SEM of three tank replicates (*n* = 3). All statistical analyses were performed using GraphPad Prism 9.0.

## 3. Results

### 3.1. Dietary SL and its Combinations Promoted Growth Performance

The growth performance of longsnout catfish is shown in Table 3. Two‐way ANOVA revealed significant sex × dietary group interactions in FBW, WGR, and SGR (*p* < 0.05). Simple main effects analysis showed that in females, the SI group exhibited significantly higher values for these growth parameters than all other groups, and the SC group was significantly higher than the control group (*p* < 0.05). In males, the SC and SI groups were significantly higher than the control and SL groups (*p* < 0.05). Regarding sex differences, males showed significantly higher growth than females in all cases except the control group for WGR and SGR. Among indices without significant interaction, GSI was significantly higher in females than in males, and the SC and SI groups showed significantly higher GSI than the SL group, which in turn was significantly higher than the control group (*p* < 0.05). VSI was significantly higher in males than in females, and the SC and SI groups were significantly higher than the control and SL groups. HSI and CF were affected only by diet, with HSI significantly lower in the SC and SI groups than in the control group and CF significantly higher in the SI group than in the control group. IBW was significantly higher in males than in females, with no significant differences among dietary groups. The survival rate showed no significant differences among all groups.

**Table 3:** Effects of different dietary additives on the growth performance of longsnout catfish.

Item	IBW (g)	FBW (g)	WGR (%)	SGR (%/d)	GSI (%)	VSI (%)	HSI (%)	CF (g/cm^3^)	Survival rate (%)
F‐control	342.57 ± 4.57	609.64 ± 14.16^a^ ^∗^	77.92 ± 2.10^a^	0.64 ± 0.01^a^	0.47 ± 0.01	5.87 ± 0.24	1.07 ± 0.05	1.37 ± 0.03	95.56 ± 2.22
F‐SL	348.73 ± 3.73	649.58 ± 9.61^ab^ ^∗^	86.25 ± 0.92^ab^ ^∗^	0.69 ± 0.01^ab^ ^∗^	0.54 ± 0.01	6.09 ± 0.09	0.97 ± 0.02	1.39 ± 0.02	97.78 ± 2.22
F‐SC	344.67 ± 3.18	673.10 ± 10.15^b^ ^∗^	95.31 ± 3.20^b^ ^∗^	0.74 ± 0.02^b^ ^∗^	0.63 ± 0.02	7.69 ± 0.35	0.94 ± 0.04	1.43 ± 0.05	95.56 ± 2.22
F‐SI	345.10 ± 2.58	733.27 ± 6.97^c^ ^∗^	112.53 ± 3.44^c^ ^∗^	0.84 ± 0.01^c^ ^∗^	0.62 ± 0.02	8.06 ± 0.17	0.93 ± 0.02	1.47 ± 0.03	97.78 ± 2.22
M‐control	416.63 ± 4.06	745.33 ± 9.51^x^	78.91 ± 2.19^x^	0.65 ± 0.01^x^	0.33 ± 0.01	6.46 ± 0.20	1.00 ± 0.01	1.38 ± 0.03	93.33 ± 3.85
M‐SL	417.01 ± 3.75	827.97 ± 11.28^y^	98.54 ± 1.66^y^	0.76 ± 0.01^y^	0.37 ± 0.02	6.72 ± 0.21	0.92 ± 0.05	1.41 ± 0.02	97.78 ± 2.22
M‐SC	418.21 ± 2.72	905.01 ± 10.18^z^	116.45 ± 3.76^z^	0.86 ± 0.02^z^	0.47 ± 0.01	8.03 ± 0.16	0.89 ± 0.04	1.46 ± 0.03	100.00 ± 0.00
M‐SI	418.07 ± 3.72	938.40 ± 9.15^z^	124.50 ± 3.13^z^	0.90 ± 0.02^z^	0.46 ± 0.02	8.39 ± 0.07	0.91 ± 0.02	1.48 ± 0.02	95.56 ± 2.22
Main effect (diet)									
Control	379.60	NA	NA	NA	0.40^a^	6.17^a^	1.03^b^	1.37^a^	94.44
SL	382.87	NA	NA	NA	0.46^b^	6.41^a^	0.94^ab^	1.40^ab^	97.78
SC	381.44	NA	NA	NA	0.55^c^	7.86^b^	0.92^a^	1.45^ab^	97.78
SI	381.59	NA	NA	NA	0.54^c^	8.23^b^	0.92^a^	1.48^b^	96.67
Main effect (sex)									
Female	345.27^A^	NA	NA	NA	0.57^B^	6.93^A^	0.98	1.42	96.67
Male	417.48^B^	NA	NA	NA	0.41^A^	7.40^B^	0.93	1.43	96.67
*p*‐Value (two‐way ANOVA)									
Diet	0.84	<0.01	<0.01	<0.01	<0.01	<0.01	0.01	0.01	0.47
Sex	<0.01	<0.01	<0.01	<0.01	<0.01	<0.01	0.07	0.41	>0.99
Diet × sex	0.84	<0.01	0.02	0.01	0.80	0.82	0.86	0.99	0.47

*Note:* Main‐effect means are not presented for FBW, WGR, and SGR because of a significant diet × sex interaction (*p* < 0.05). Values are mean of three tank replicates (*n* = 3). Data are expressed as mean ± SEM. For variables with a significant diet × sex interaction, different lowercase letters (a, b, c, and d for females; x, y, z, and w for males) indicate significant differences among dietary groups within each sex (*p* < 0.05), and asterisks ( ^∗^) placed on female means indicate significant differences between sexes within the same dietary group (*p* < 0.05). For variables without a significant interaction, different lowercase letters indicate significant differences among dietary groups pooled across sexes (*p* < 0.05), and different uppercase letters indicate significant differences between sexes pooled across diets (*p* < 0.05). Sharing the same letter indicates no significant difference. The same applies as below.

Abbreviations: CF, condition factor; F, female; FBW, final body weight; GSI, gonadosomatic index; HSI, hepatosomatic index; IBW, initial body weight; M, male; NA, not applicable; SGR, specific growth rate; VSI, viscerosomatic index; WGR, weight gain rate.

### 3.2. Dietary SL and its Combinations Promoted Ovarian Development

**Figure 1:**
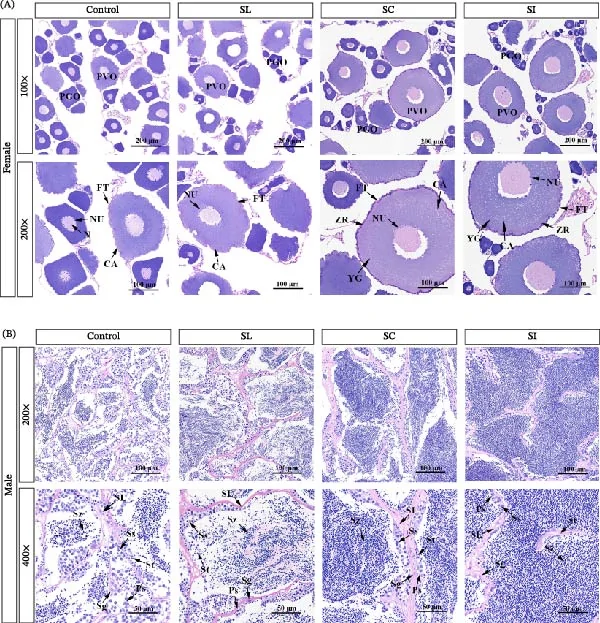
Effects of dietary additives on the gonadal development in longsnout catfish. (A) Hematoxylin and eosin (HE) sections of ovaries from control, SL, SC, and SI groups. CA, cortical alveoli; FT, follicular theca; N, nucleus; NU, nucleolus; PGO, primary growth stage oocyte; PVO, previtellogenin oocyte; YG, yolk granule; ZR, zona radiate. (B) HE sections of testes from control, SL, SC, and SI groups. Ps, primary spermatocytes; Sg, spermatogonia; SL, seminiferous lobules; Ss, secondary spermatocytes; St, spermatids; Sz, spermatozoa.

Histological observations revealed (Figure 1A) that in the control group, the ovaries primarily contained primary growth oocytes (PGOs), which were smaller in size, densely arranged, exhibited strong basophilic staining with deeply stained cytoplasm, and displayed several nucleoli around the nuclear membrane, with an indistinct outer follicular theca. Compared with the control group, the volume of PGOs in the ovaries of the SL group increased appreciably. Most PGOs formed a distinct follicle theca externally, which was composed of follicle cells, and cortical alveoli were visible along the inner edges of the cells. In the SC and SI groups, some previtellogenin oocytes (PVOs) were observed. The cytoplasm of these PVOs presented an increased number of cortical alveolar cells, with yolk granules beginning to appear and a marked increase in cell volume. The follicle theca transformed from a single layer to a double‐layered structure, and a distinct zona radiata emerged between the cell membrane and the follicular cells.

In the testes of the control group (Figure 1B), the seminiferous lobules contained abundant connective tissue and various stages of spermatogenic cells, primarily consisting of primary spermatocytes (Ps) and secondary spermatocytes (Ss). In the SL group, the number of spermatozoa (Sz) increased, the spermatozoa volume was smaller than that of spermatids, and the nuclei showed stronger basophilia with deeper staining. In the SC and SI groups, the testes were mainly filled with a large number of spermatozoa. Some of the connected capsule walls fused, the seminiferous lobules expanded, and the number of sperm in the seminiferous lobules increased, showing more synchronous development.

In Table 4, the PVO diameter in females was significantly higher in the SC and SI groups than in the control and SL groups (*p* < 0.05). PGO diameter, PGO percentage, and PVO percentage did not differ significantly among groups. In males, sperm density was significantly higher in the SC and SI groups than in the control groups (*p* < 0.05). The percentages of spermatogonia, spermatocytes, and spermatids were all significantly lower in the three treatment groups than in the control group (*p* < 0.05), with no differences among treatments. Spermatozoa percentage was significantly higher in the SC and SI groups than in the SL group, and the SL group was significantly higher than the control group (*p* < 0.05).

**Table 4:** Effects of different dietary additives on gonadal histological parameters in female and male longsnout catfish.

Parameter	Control	SL	SC	SI
Female				
PGO diameter (µm)	79.19 ± 6.65	68.78 ± 3.83	76.71 ± 6.03	71.44 ± 6.61
PVO diameter (µm)	162.01 ± 2.40^a^	171.22 ± 3.61^a^	296.57 ± 17.41^b^	292.47 ± 14.14^b^
PGO (%)	74.18 ± 2.60	66.00 ± 2.69	59.81 ± 8.07	61.91 ± 3.70
PVO (%)	25.82 ± 2.60	34.00 ± 2.69	40.19 ± 8.07	38.09 ± 3.70
Male				
Sperm density (×10^5^ cells/mm^2^)	6.61 ± 0.78^a^	9.72 ± 0.15^ab^	14.33 ± 0.73^c^	12.99 ± 1.27^bc^
Spermatogonia (%)	5.09 ± 1.08^b^	1.35 ± 0.15^a^	0.43 ± 0.04^a^	0.63 ± 0.06^a^
Spermatocytes (%)	13.06 ± 2.01^b^	6.64 ± 0.86^a^	2.33 ± 0.60^a^	2.74 ± 0.90^a^
Spermatids (%)	15.57 ± 2.70^b^	6.54 ± 1.03^a^	3.08 ± 0.38^a^	3.18 ± 0.90^a^
Spermatozoa (%)	66.27 ± 1.57^a^	85.47 ± 1.61^b^	94.16 ± 0.98^c^	93.45 ± 1.82^c^

*Note:* Different lowercase letters (a, b, c, d) indicate significant differences among groups in the same row (*p* < 0.05).

Abbreviations: PGO, primary growth stage oocyte; PVO, previtellogenic oocyte.

### 3.3. Dietary SL and Its Combination Regulated Reproductive Hormones

**Figure 2:**
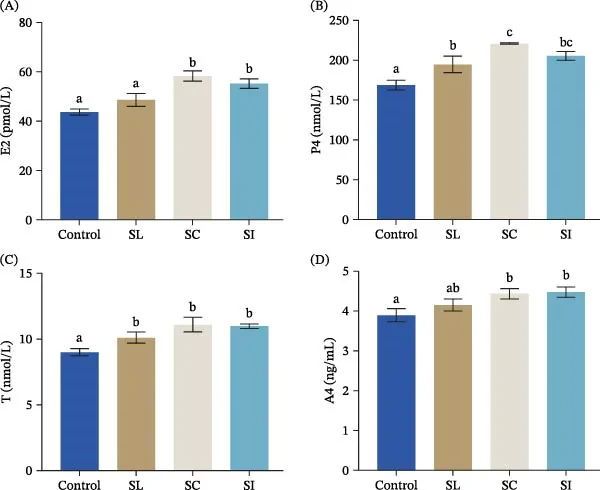
Effects of different dietary additives on serum hormone levels in longsnout catfish. (A) Serum 17β‐estradiol (E2) levels in females; (B) serum progesterone (P4) levels in females; (C) serum testosterone (T) levels in males; and (D) serum androstenedione (A4) levels in males. Values are mean ± SEM of three tank replicates (*n* = 3); means with different superscripts within a column differ significantly (*p* < 0.05). The same applies as below.

In female fish, serum E2 levels were markedly elevated in the SC and SI groups relative to the control and SL groups (*p* < 0.05), with no significant difference between the control and SL groups. P4 levels were significantly increased in all three treatment groups compared with the control, and the SC group exhibited higher P4 levels than the SL group (*p* < 0.05) (Figure 2A, B).

In male fish, T levels were significantly higher in all treatment groups than in the control group (*p* < 0.05), while no significant differences were observed among the three treatment groups. A4 levels were also significantly elevated in the SC and SI groups relative to the control group (*p* < 0.05), with no significant differences among the other groups (Figure 2C, D).

### 3.4. Dietary SL and its Combination‐Promoted Antioxidant and Immune Capabilities

As shown in Table 5, two‐way ANOVA revealed no significant sex × dietary interactions for T‐AOC, SOD, CAT, AKP, MDA, or cortisol. Diet significantly affected all indices. T‐AOC activity was significantly higher in the SC and SI groups than in the control and SL groups (*p* < 0.05), with no difference between SC and SI or between control and SL. SOD activity was highest in the SI group, significantly higher than all other groups, and the SC group was significantly higher than the control (*p* < 0.05). CAT activity was significantly higher in SL, SC, and SI compared to control (*p* < 0.05), with no differences among the three treated groups. AKP activity was highest in SI, significantly higher than SL, SC, and control, while SL and SC were also higher than the control (*p* < 0.05). MDA content was significantly reduced in SC and SI than in SL and control, and SL was lower than control (*p* < 0.05). Cortisol levels were significantly reduced in SC and SI compared to control, and SI was lower than SL (*p* < 0.05). Regarding sex, SOD, CAT, and cortisol were significantly higher in females than in males, whereas AKP was higher in males (*p* < 0.05); T‐AOC and MDA showed no significant sex differences.

**Table 5:** Effects of different dietary additives on oxidative stress indices in longsnout catfish.

Item	T‐AOC (nmol Trolox/mL)	SOD (U/mL)	CAT (μmol/min/mL)	AKP (×10^−2^ μmol/min/mL)	MDA (nmol/mL)	Cortisol (ng/mL)
F‐control	135.74 ± 9.25	6.79 ± 0.06	19.50 ± 1.08	3.83 ± 0.43	4.16 ± 0.34	3005.79 ± 37.45
F‐SL	134.47 ± 4.12	7.34 ± 0.31	22.26 ± 1.20	4.94 ± 0.29	2.96 ± 0.21	2909.80 ± 69.27
F‐SC	155.67 ± 4.92	8.64 ± 0.50	24.49 ± 0.77	5.74 ± 0.49	2.36 ± 0.23	2643.90 ± 83.99
F‐SI	172.20 ± 9.15	9.15 ± 0.33	24.22 ± 1.71	6.54 ± 0.47	2.60 ± 0.23	2777.21 ± 91.19
M‐control	127.79 ± 3.29	6.37 ± 0.30	17.94 ± 0.42	4.64 ± 0.41	4.37 ± 0.31	2931.41 ± 48.84
M‐SL	144.66 ± 4.11	7.21 ± 0.26	21.95 ± 0.49	6.54 ± 0.55	3.94 ± 0.17	2698.75 ± 41.09
M‐SC	168.39 ± 10.83	7.17 ± 0.38	22.56 ± 1.47	6.63 ± 0.60	2.31 ± 0.16	2488.88 ± 19.55
M‐SI	161.54 ± 7.47	8.85 ± 0.25	21.39 ± 0.85	9.30 ± 0.70	2.58 ± 0.14	2696.20 ± 84.20
Main effect (diet)						
Control	131.77^a^	6.58^a^	18.72^a^	4.24^a^	4.26^c^	2968.60^c^
SL	139.57^a^	7.28^ab^	22.11^b^	5.74^b^	3.45^b^	2804.27^bc^
SC	162.03^b^	7.90^b^	23.52^b^	6.18^b^	2.34^a^	2566.39^a^
SI	166.87^b^	9.00^c^	22.81^b^	7.92^c^	2.59^a^	2736.70^ab^
Main effect (sex)						
Female	149.52	7.98^B^	22.62^B^	5.26^A^	3.02	2834.17^B^
Male	150.59	7.40^A^	20.96^A^	6.78^B^	3.30	2703.81^A^
*p*‐Value (two‐way ANOVA)						
Diet	<0.01	<0.01	<0.01	<0.01	<0.01	<0.01
Sex	0.84	0.02	0.05	<0.01	0.11	0.01
Diet × sex	0.27	0.19	0.71	0.23	0.14	0.68

*Note:* For variables without a significant interaction, different lowercase letters (a, b, c, d) denote significant differences among dietary groups pooled across sexes (*p* < 0.05), and different uppercase letters (A, B) denote significant differences between sexes pooled across diets (*p* < 0.05). Shared letters indicate no significant difference.

Abbreviations: AKP, alkaline phosphatase; CAT, catalase; MDA, malondialdehyde; SOD, superoxide dismutase; T‐AOC, total antioxidant capacity.

### 3.5. Intestinal Microbiota Composition

Quality control statistics and ASV counts for all samples (3 tanks × 4 treatments × 2 sexes) are presented in Table S1. In Table 6, one‐way ANOVA revealed no significant differences among any of the alpha‐diversity indices after FDR correction. Good’s coverage showed no significant differences among groups, and the rarefaction curves plateaued with increasing sequencing depth (Figure 3A). PCoA (Figure 3B) revealed that PC1 and PC2 explained 37.94% and 18.64% of the total variation, respectively. Samples in the SC and SI groups clustered tightly, indicating good within‐group reproducibility. The SL and control groups showed greater within‐group dispersion and substantial overlap between them. The SL group had a wider distribution, and there was a slight overlap between the control group and the SC/SI clusters.

Figure 3Effects of different dietary treatments on the intestinal microbiota composition of longsnout catfish. (A) Rarefaction curve of Good’s coverage; (B) principal coordinate analysis (PCoA) analysis in the control, SL, SC, and SI groups; (C) relative abundance of bacterial phyla; (D) relative abundance of bacterial genera; (E) box plot of Bacteroidota abundance in females; (F) box plots of *Bacteroides* and *Plesiomonas* abundance in females; and (G) box plots of *Bacteroides* and *Cetobacterium* abundance in males. Different lowercase letters within each box plot indicate significant differences among groups (Tukey’s HSD post‐hoc test, adjusted *p* < 0.05).

**Figure:**
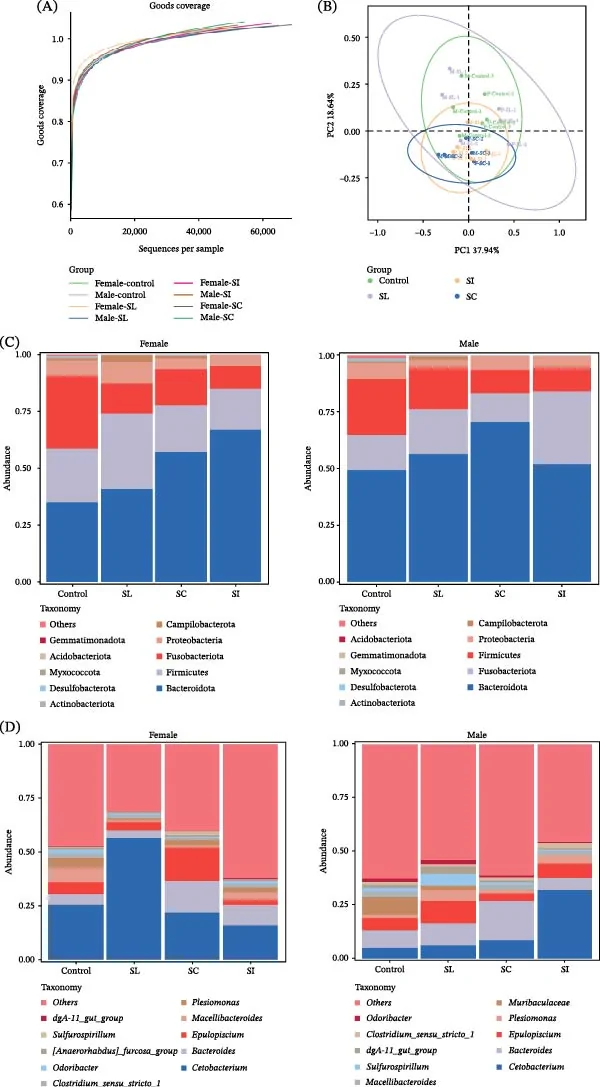

**Figure:**
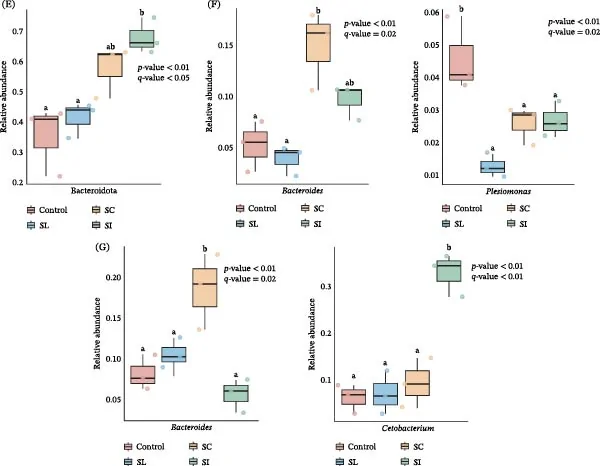

**Table 6:** Effects of different dietary additives on the *α*‐diversity of intestinal microbiota of longsnout catfish.

Item	PD	Chao1	Shannon	Simpson	Observed species	ACE
F‐control	19.46 ± 1.37	180.96 ± 9.45	4.34 ± 0.06	0.87 ± 0.01	178.77 ± 8.41	182.44 ± 9.04
F‐SL	21.54 ± 2.04	180.92 ± 18.61	4.34 ± 0.22	0.91 ± 0.02	179.33 ± 19.34	180.23 ± 18.42
F‐SC	22.77 ± 1.78	193.63 ± 15.10	4.44 ± 0.02	0.90 ± 0.01	191.27 ± 15.69	190.28 ± 16.52
F‐SI	21.75 ± 0.85	190.18 ± 10.89	5.04 ± 0.14	0.94 ± 0.01	188.93 ± 10.33	189.95 ± 11.10
*p*‐Value	0.54	0.88	0.02	0.02	0.89	0.94
*q*‐Value	0.94	0.94	0.07	0.07	0.94	0.94
M‐control	19.38 ± 0.50	167.06 ± 14.79	4.55 ± 0.43	0.90 ± 0.03	164.93 ± 14.84	170.81 ± 16.14
M‐SL	24.81 ± 0.36	209.72 ± 13.60	5.20 ± 0.16	0.93 ± 0.01	206.90 ± 12.75	208.40 ± 13.19
M‐SC	24.92 ± 2.95	235.46 ± 55.28	5.43 ± 0.30	0.95 ± 0.01	232.87 ± 53.32	235.18 ± 56.45
M‐SI	27.43 ± 1.98	244.04 ± 29.09	5.02 ± 0.23	0.93 ± 0.01	240.20 ± 27.66	250.94 ± 33.07
*p*‐Value	0.07	0.40	0.27	0.27	0.38	0.42
*q*‐Value	0.47	0.50	0.50	0.50	0.50	0.50

Abbreviations: ACE, abundance‐based coverage estimator; PD, phylogenetic diversity.

As shown in Figure 3C, Bacteroidota, Firmicutes, Fusobacteriota, and Proteobacteria were the dominant phyla in the intestinal microbiota. Post‐hoc multiple comparison tests were performed on taxa with a *q*‐value < 0.05. The results showed that in females, the relative abundance of Bacteroidota in the SI group was significantly higher than that in the control and SL groups (adjusted *p* < 0.05) (Figure 3E).

As shown in Figure 3D, *Cetobacterium*, *Bacteroides*, *Epulopiscium*, *Macellibacteroides*, and *Plesiomonas* were the dominant genera in the intestinal microbiota. Post‐hoc multiple comparison tests on taxa with *q* < 0.05 revealed that in females, the relative abundance of *Bacteroides* in the SC group was significantly increased compared to the control and SL groups, while *Plesiomonas* in the control group showed significantly higher relative abundance than in all other groups (adjusted *p* < 0.05) (Figure 3F). In males, the relative abundance of *Bacteroides* in the SC group was significantly higher than in all other groups, and *Cetobacterium* in the SI group was also significantly elevated compared with the other groups (adjusted *p* < 0.05) (Figure 3G).

### 3.6. Transcriptome Analysis

Transcriptome analysis was performed separately on ovaries from females and testes from males. A total of 24 cDNA libraries were constructed (3 tanks × 4 treatments × 2 sexes). As shown in Table S2, a total of 24 cDNA libraries were constructed. The number of clean bases per sample ranged from 10.04 M to 11.76 M, and the Q30 base rate was higher than 97.10%. The obtained clean reads were aligned to the reference genome of longsnout catfish, with alignment rates ranging from 89.54% to 93.35% among the libraries.

The identified DEGs from pairwise comparisons in both sexes were presented in volcano plots (Figure S2), and the shared and unique DEGs among different comparison groups are shown in Figure S3. In females, a total of 14 DEGs (12 upregulated and 2 downregulated) were identified in SL vs. control, 21 DEGs (16 upregulated and 5 downregulated) were identified in SC vs. control, and 12 DEGs (7 upregulated and 5 downregulated) were identified in SI vs. control. In males, a total of 41 DEGs (6 upregulated, 35 downregulated), 189 DEGs (37 upregulated, 152 downregulated), and 86 DEGs (8 upregulated, 78 downregulated) were identified in SL vs. control, SC vs. control, and SI vs. control, respectively.

Based on the identified DEGs, GO and KEGG pathway enrichment analyses were further conducted. The top 10 significantly enriched GO terms (*q* < 0.05) of DEGs for each sex and dietary comparison group are shown in Figure S4. Among these, most target DEGs were enriched in terms belonging to the “biological process” category, while a few were enriched in the “molecular function” and “cellular component” categories.

**Figure 4:**
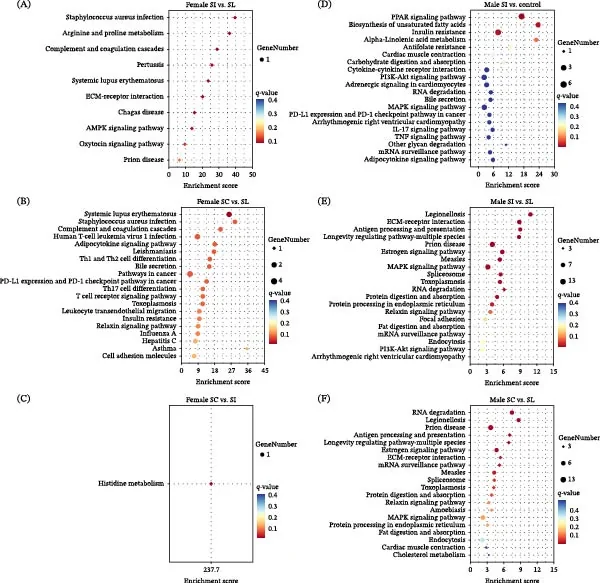
Kyoto Encyclopedia of Genes and Genomes (KEGG) enrichment analysis of DEGs. (A) SI vs. SL group in females; (B) SC vs. SL group in females; (C) SC vs. SI group in females; (D) SI vs control group in males; (E) SI vs. SL group in males; and (F) SC vs. SL group in males.

The significantly enriched KEGG pathways among pairwise comparisons in both genders are shown in Figure 4. In females, the systemic lupus erythematosus pathway was significantly enriched in the SC vs. SL comparison (*q* < 0.05); DEGs were significantly enriched in the histidine metabolism pathway in the SC vs. SI comparison (*q* < 0.05). In males, in the SI vs. control comparison, significantly enriched pathways included the peroxisome proliferator‐activated receptor (PPAR) signaling pathway, biosynthesis of unsaturated fatty acids, and insulin resistance. In the SC vs. SL comparison, DEGs were significantly enriched in 11 pathways (*q* < 0.05), including the estrogen signaling pathway, antigen processing and presentation, and RNA degradation. In the SI vs. SL comparison, DEGs were significantly enriched in 13 pathways (*q* < 0.05), including the estrogen signaling pathway, the mitogen‐activated protein kinase (MAPK) signaling pathway, and protein digestion and absorption. KEGG enrichment results for the remaining comparison groups showed no significance and are presented in Figure S5.

**Figure 5:**
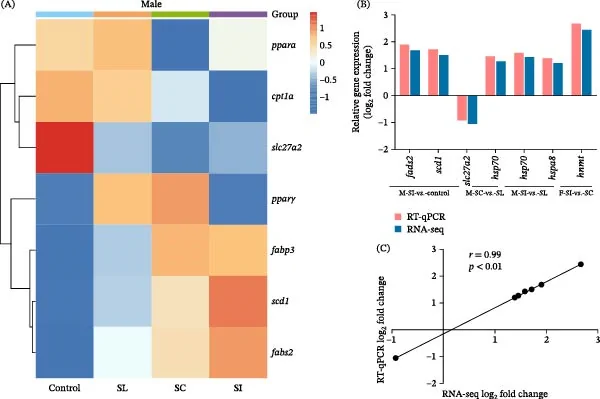
RNA‐seq validation and peroxisome proliferator‐activated receptor (PPAR) pathway gene expression. (A) Heatmap of the expression of PPAR pathway genes in males; (B) validation of the RNA sequencing data using RT‐qPCR; and (C) Pearson correlation between log_2_ fold changes of RNA‐seq and RT‐qPCR. RT‐qPCR, reverse transcription quantitative real‐time PCR; F, female; M, male.

Through *Z*‐score normalization, heatmaps of key genes in the PPAR signaling pathway were generated (Figure 5A). The expression of fatty acid desaturase 2 (*fads2*), stearoyl‐CoA desaturase 1 (*scd1*), and solute carrier family 27 member 2 (*slc27a2*) was significantly altered in the SI group compared with the control group (*q* < 0.05). In contrast, the expression changes of PPAR alpha (*pparα*), PPAR gamma (*pparγ*), carnitine palmitoyltransferase 1A (*cpt1a*), and fatty acid binding protein 3 (*fabp3*) did not reach statistical significance.

### 3.7. Validation of the RNA‐seq Results by RT‐qPCR

As shown in Figure 5B, the expression patterns of the selected DEGs obtained by RT‐qPCR were consistent with those from RNA‐seq. Pearson correlation analysis between the log_2_ fold changes of RNA‐seq and RT‐qPCR revealed a significant positive correlation, confirming the reliability of the transcriptome data (Figure 5C).

## 4. Discussion

Due to the beneficial effects of functional feed additives, increasing numbers of studies have focused on their potential applications in aquaculture diets. Research has shown that fish cannot synthesize sufficient phospholipids and therefore require dietary supplementation to enhance growth performance and promote sexual maturation [10, 30]. Studies on silvery‐black porgy (*Sparidentex hasta*) and yellowtail (*Seriola quinqueradiata*) have shown that dietary SL supplementation effectively improves growth performance [31, 32]. In the present study, three treatment groups significantly improved WGR and SGR in males, whereas in females, this improvement was only observed in the SC and SI groups. Moreover, growth performance in females was further enhanced in the SI group compared to the SC group, suggesting that INO, as a metabolic modulator, may have greater potential than VC when combined with SL to promote growth. Notably, this growth‐promoting effect was more pronounced in males than in females.

HSI reflects the energy reserve status in the liver of fish, while GSI serves as an indicator of their reproductive status. Phospholipids enhance lipid metabolism, transport, and deposition in fish, and the liver is the primary organ involved in lipid metabolism [30]. In this study, HSI was significantly lower in the SC and SI groups than in the control group, and all treated groups exhibited significantly higher GSI compared with the control group, suggesting that combined supplementation with INO may promote lipid mobilization from the liver to the gonads during sexual maturation. A possible explanation is that during the gonadal maturation stage, lipids stored in the liver are mobilized to the gonads to meet the high energy demands of reproduction [33, 34]. This finding is also consistent with the study on rice field eel (*Monopterus albus*), which showed that dietary SL supplementation promoted ovarian development by influencing the activity of lipid metabolism‐related enzymes and the secretion of sex hormones but reduced the HSI [33]. Interestingly, the enhanced gonadal development observed in the SC group may be partly attributed to improved hepatic lipid metabolism, whereby VC helps maintain normal liver function and facilitates the mobilization of lipids to the gonads during sexual maturation rather than simply reducing hepatic lipid storage.

The present study demonstrated that dietary supplementation with SL promoted gonadal development in both female and male longsnout catfish, as evidenced by increased oocyte volume, active spermatogenesis, and elevated levels of sex steroid hormones (E2, P4, T, and A4). Sex steroid hormones are critical regulators of germ cell development: E2 controls vitellogenin synthesis and ovarian development, while T, as the primary androgen in fish, is closely associated with testicular development [34–36]. The concomitant elevation of these hormones in SL‐fed fish suggests that SL may modulate steroidogenesis by altering the phospholipid composition of germ cell membranes, thereby influencing membrane‐mediated gonadotropin receptor sensitivity [33, 37].

Notably, the combination of SL with VC or INO further enhanced sex hormone secretion and gonadal maturation. In females, the SC and SI groups exhibited significantly larger PVO diameters than the control groups. In males, the SC group achieved the highest sperm density, significantly exceeding the control and SL groups, while the SI group also showed a higher sperm density than the control groups. This enhanced effect observed with combined supplementation may be attributed to the multiple physiological functions of VC, which serves as a cosubstrate for hydroxylases and oxygenases involved in the biosynthesis of procollagen, carnitine, and neurotransmitters, as well as antioxidant or prooxidant activities—processes essential for tissue development and metabolic function [7, 38]. Studies on Japanese eel (*Anguilla japonica*) [39] and Nile tilapia [7] have demonstrated that VC supplementation improves reproductive performance, likely through its antioxidant protection of developing gametes.

AKP is a nonspecific immune‐related enzyme, while CAT, SOD, and T‐AOC are key components of the antioxidant defense system in aquatic animals, with changes in their activity reflecting the organism’s health status [40]. Moreover, MDA is the end product of lipid peroxidation, and its prolonged elevation indicates tissue damage [41, 42]. Cortisol is the primary stress hormone, and its prolonged elevation suppresses immune function, impedes growth, and interferes with reproductive processes [43, 44]. In this study, dietary SL supplementation significantly reduced MDA content, indicating attenuated lipid peroxidation but did not significantly alter antioxidant enzyme activities or cortisol levels. In contrast, combined supplementation with VC or INO significantly enhanced antioxidant enzyme activities and T‐AOC and reduced cortisol levels. Conversely, studies on common carp (*Cyprinus carpio*) and Caspian brown trout (*Salmo trutta caspius*) revealed that SL supplementation significantly enhanced antioxidant capacity [45, 46]. This discrepancy may be attributed to differences in SL supplementation levels or species‐specific responses, warranting further investigation. Interestingly, the present study showed that the combined supplementation of SL with VC or INO significantly improved antioxidant and stress‐protective capacities, consistent with previous findings [47].

The intestine is a complex organ in aquatic animals, and the gut microbiota plays a crucial role in nutrient digestion, metabolism, and immune responses [48]. Diet composition can significantly influence the structure of the intestinal microbiota in fish [49]. Dominant phyla in aquatic animals generally include Bacteroidota, Firmicutes, and Proteobacteria, among others [50, 51]. Proteobacteria are often associated with diseases as they include various pathogenic bacteria, such as *Escherichia*, *Salmonella*, and *Rickettsia* [52]. Bacteroidota can break down indigestible dietary polysaccharides, providing energy for the host while inhibiting pathogen colonization and maintaining intestinal homeostasis [53, 54]. In this study, dietary SI significantly increased the relative abundance of Bacteroidota in the gut of female fish, while the abundance of Proteobacteria was not significantly affected by the dietary additives, which suggested that SI may promote a more favorable gut microbial composition, potentially through improved intestinal digestion.

At the genus level, SC significantly increased the abundance of Bacteroides in both female and male fish, while the abundance of the potentially pathogenic genus Plesiomonas was significantly reduced in all three treatment groups of females. Additionally, SI increased the abundance of *Cetobacterium* in males. *Cetobacterium* is a potentially beneficial bacterium commonly found in the fish gut, with roles in vitamin B12 production and carbohydrate metabolism [55]. *Bacteroides* are also considered potentially beneficial, contributing to polysaccharide degradation and intestinal homeostasis [56, 57]. In contrast, *Plesiomonas* includes potentially pathogenic species associated with fish diseases [58]. In summary, dietary SL, SC, and SI may improve gut microbial community composition by promoting the growth of potentially beneficial genera and inhibiting the colonization of potentially harmful genera, with SC and SI generally showing stronger effects than SL alone. However, the specific functions of these taxa were still not revealed in this study, and further validation using metagenomic or culture‐based approaches should be performed in the future.

In this study, transcriptomic analysis was performed to explore how SL, VC, and INO exert positive effects on growth, antioxidant capacity, and gonad development. GO terms and KEGG pathways indicated that the three functional additives may have similar effects on fish at the transcriptomic level, but their influence appears to be greater in males than in females.

In males, both the PPAR signaling pathway and the biosynthesis of unsaturated fatty acid pathway were significantly enriched in the SI vs. control comparison. These two pathways shared key DEGs *fads2* and *scd1*, both of which were significantly upregulated in the SI group. FADS2 is a key enzyme for the synthesis of long‐chain polyunsaturated fatty acids (LC‐PUFAs) [59, 60], while SCD1 catalyzes the conversion of saturated fatty acids to monounsaturated fatty acids [61]. Their upregulation suggested that SL combined with INO enhanced endogenous fatty acid desaturation in the gonads. Studies have shown that SCD1 and FADS2 contribute to the maintenance of cellular redox homeostasis during lipid accumulation [62]. In contrast, the fatty acid transport‐related gene *slc27a2* within the PPAR pathway was significantly downregulated in the SI group. The SLC27 family mediates the transmembrane transport of long‐chain fatty acids [63], which suggested that gonadal cells underwent metabolic regulation, potentially reducing their dependence on exogenous fatty acids while shifting toward enhanced endogenous fatty acid desaturation. The significant changes in downstream target genes such as *fads2*, *scd1*, and s*lc27a2* suggested that SI treatment may regulate PPAR signaling through downstream target genes. Moreover, the estrogen signaling pathway was significantly enriched in both the SC vs. SL and SI vs. SL comparisons, with heat shock protein 70 (*hsp70*) significantly upregulated in both the SC and SI groups and heat shock protein family A member 8 (*hspa8*) additionally upregulated in the SI group. HSP70 and HSPA8 were members of the heat shock protein 70 family [64, 65]. As ATP‐dependent molecular chaperones, they assisted the estrogen receptor (ER) in maintaining its correct conformation in the cytoplasm and promoted its binding to estrogen. More importantly, their primary function lay in assisting proper protein folding, preventing protein aggregation, and maintaining intracellular protein homeostasis [64, 66, 67]. During spermatogenesis, germ cells undergo rapid proliferation and differentiation, accompanied by elevated protein synthesis and increased oxidative stress, making this chaperone function particularly critical [68, 69]. In the testis of the teleost *Prochilodus argenteus*, HSP70 expression increases during the sperm maturation stage and may protect germ cells by inhibiting caspase‐3‐dependent apoptosis, thereby maintaining normal spermatogenesis [70]. Notably, HSP70 is stress‐inducible, whereas HSPA8 is a constitutively expressed member, and their coordinated upregulation in the testis of the SI group may provide complementary protection [71]. Therefore, the upregulation of *hsp70* and *hspa8* within the estrogen signaling pathway suggested that SC and SI treatments may enhance the chaperone response associated with estrogen signaling, assisting germ cells in coping with protein synthesis and oxidative stress, thereby providing protection for spermatogenesis.

In females, the histidine metabolism pathway was significantly enriched in the SC vs. SI comparison, with the key gene *N*‐methyltransferase (*hnmt*) upregulated in the SC group relative to SI. HNMT is the key enzyme that catalyzes the *N*‐methylation inactivation of histamine, converting histamine to *N*‐methylhistamine and thereby abolishing its biological activity [72, 73]. It was revealed that in teleost fish, histamine acted as an important proinflammatory mediator and regulated the inflammatory response by acting on professional phagocytes [74]. The transcriptional upregulation of *hnmt* in the ovaries of female fish in the SC group may reflect a regulatory mechanism that prevents excessive histamine accumulation in the ovarian environment by enhancing histamine inactivation capacity, potentially contributing to the maintenance of local ovarian immune homeostasis [75, 76]. This female‐specific functional difference suggested that the combined use of SL and VC may offer unique immunomodulatory advantages in female broodstock nutrition, although this inference requires further functional validation. Taken together, the combined application of SL with VC or INO may promote gonadal development by influencing multiple aspects, including lipid metabolism, estrogen signaling, and immune homeostasis. However, the transcriptomic data were derived solely from gonadal tissues; whether this lipid accumulation was supported by hepatic lipid mobilization required further investigation.

It also should be noted that we just assessed the combined application of SL with VC or INO, but separate VC and INO groups were not included. The beneficial effects observed in the SC and SI groups are interpreted as resulting from combined supplementation with SL rather than from VC or INO alone. Future investigations using a full factorial design would be needed to further clarify their separate and interactive contributions.

## 5. Conclusion

The SI group showed the best growth‐promoting effect in females, while both SC and SI were effective in promoting gonadal maturation, antioxidant capacity, and intestinal microbiota composition in both male and female fish. Dietary SI and SC increased potentially beneficial *Bacteroides* in both sexes. SI increased potentially beneficial *Cetobacterium* in males, and potentially pathogenic *Plesiomonas* decreased in all treated females. Gonadal transcriptome analysis indicated that these improvements may be mediated through the PPAR signaling, biosynthesis of unsaturated fatty acids, estrogen signaling, and histidine metabolism pathways. As separate VC and INO groups were not included, the enhanced effects in the SC and SI groups were attributable to the combination with SL rather than to VC or INO alone. This study provides theoretical guidance for the development of specialized combined diets during the gonadal development period in longsnout catfish.

## Author Contributions

**Ming-Hua Xie**: conceptualization, formal analysis, investigation, writing – original draft. **Yu-Lin Zhou, Min Liu, Zhi Li, Peng Zhen, Jun Tian, Yi Gong, Xiao-Juan Zhang, Dong Han, and Li Zhou**: methodology, resources. **Jian-Fang Gui and Zhong-Wei Wang**: writing – review and editing, supervision, project administration, funding acquisition.

## Funding

This work was supported by the National Key Research and Development Program of China (Grant 2022YFD2400903).

## Disclosure

All authors have read and approved the final manuscript.

## Ethics Statement

All experiments and animal treatments were approved by the Institutional Animal Care and Use Committee of the Institute of Hydrobiology (IHB2024‐0405).

## Conflicts of Interest

The authors declare no conflicts of interest.

## Supporting Information

Additional supporting information can be found online in the Supporting Information section.

## Supporting information


**Supporting Information** Figure S1: Electrophoretic results of sex identification of experimental fish in the control group. Sex‐specific primers for longsnout catfish amplify both 992 and 684 bp in males and only one fragment of 992 bp in females. M: DL 2000 DNA marker; 1–10: 10 experimental fish in the first replicate bucket of the control group. Figure S2: Volcano plot of DEGs. (A) Volcano plot of DEGs for pairwise comparisons among groups in females; (B) volcano plot of DEGs for pairwise comparisons among groups in males; red dots represent significantly upregulated DEGs; blue dots represent significantly downregulated DEGs; light pink dots represent upregulated genes that are not statistically significant; light blue dots represent downregulated genes that are not statistically significant; and gray dots represent genes with no differential expression. Figure S3: Commonalities and differences in gene expression between male and female fish across different comparison groups. F, female; M, male. Figure S4: Gene Ontology (GO) enrichment analysis of differentially expressed genes (DEGs). (A) GO enrichment analysis for pairwise comparisons among groups in females; (B) GO enrichment analysis for pairwise comparisons among groups in males. Figure S5: Kyoto Encyclopedia of Genes and Genomes (KEGG) enrichment analysis of DEGs. (A) SL vs. control group in female; (B) SC vs. control group in female; (C) SI vs. control group in female; (D) SL vs. control group in male; (E) SC vs. control group in male; and (F) SC vs. SI group in male. Table S1: Quality assessment of 16S rRNA amplicon sequencing and ASV statistics of the intestinal microbiota in longsnout catfish. Table S2: Quality assessment of transcriptome sequencing for longsnout catfish.

## Data Availability

The raw transcriptome sequencing data generated in this study have been deposited in the NCBI Sequence Read Archive under the Accession Number PRJNA1451427. Other data that support the findings of this study are available from the corresponding author upon reasonable request.
