# Supplementary material for: Combined Effects of Dietary Soybean Lecithin With Vitamin C or Inositol on Growth, Gonadal Development, and Intestinal Microbiota in Longsnout Catfish
Source: Aquac Nutr. 2026 Sep 22;2026:4061190. doi: 10.1155/anu/4061190 (PMC13595401; doi:10.1155/anu/4061190)
Supplement: Supplementary file 1 [file ANU-2026-4061190-s001.zip › Supplementary Tables-R2.docx]

**Supplementary Tables**

**Table S1 Quality assessment of 16S rRNA amplicon sequencing and ASV statistics of the intestinal microbiota in longsnout catfish.**

| **Sex** | **Samples** | **Raw Reads** | **Filtered**  **(Reads, %)** | **denoised** | **Merged**  **(Reads, %)** | **non_chimeric**  **(Reads, %)** | **ASV_counts** |
| --- | --- | --- | --- | --- | --- | --- | --- |
| **Female** | **Control-1** | 79525 | 74453(93.62%) | 72991 | 66829(84.04%) | 55586(69.90%) | 178 |
|  | **Control-2** | 80946 | 77315(95.51%) | 76451 | 73594(90.92%) | 67796(83.75%) | 199 |
|  | **Control-3** | 78021 | 74475(95.46%) | 73789 | 71153(91.20%) | 66917(85.77%) | 167 |
|  | **SL-1** | 79746 | 76057(95.37%) | 75578 | 74111(92.93%) | 73708(92.43%) | 205 |
|  | **SL-2** | 78717 | 74807(95.03%) | 74325 | 73358(93.19%) | 72529(92.14%) | 143 |
|  | **SL-3** | 78383 | 75967(96.92%) | 74747 | 69501(88.67%) | 50579(64.53%) | 193 |
|  | **SC-1** | 79050 | 75957(96.09%) | 75360 | 73558(93.05%) | 69955(88.49%) | 209 |
|  | **SC-2** | 80757 | 77571(96.05%) | 76872 | 74502(92.25%) | 69641(86.24%) | 162 |
|  | **SC-3** | 78011 | 74705(95.76%) | 74082 | 72281(92.65%) | 70437(90.29%) | 208 |
|  | **SI-1** | 78674 | 73649(93.61%) | 72852 | 69368(88.17%) | 66566(84.61%) | 196 |
|  | **SI-2** | 80297 | 76814(95.66%) | 76154 | 72965(90.87%) | 62930(78.37%) | 169 |
|  | **SI-3** | 78467 | 75251(95.90%) | 74433 | 71196(90.73%) | 63579(81.03%) | 205 |
| **Male** | **Control-1** | 79269 | 75723(95.53%) | 74935 | 71267(89.91%) | 63752(80.42%) | 187 |
|  | **Control-2** | 79803 | 76353(95.68%) | 75566 | 72590(90.96%) | 63813(79.96%) | 175 |
|  | **Control-3** | 78517 | 75477(96.13%) | 74751 | 71751(91.38%) | 63165(80.45%) | 137 |
|  | **SL-1** | 78962 | 75502(95.62%) | 75042 | 73251(92.77%) | 71165(90.13%) | 196 |
|  | **SL-2** | 80303 | 77182(96.11%) | 76638 | 74325(92.56%) | 72541(90.33%) | 235 |
|  | **SL-3** | 78950 | 75848(96.07%) | 75137 | 72084(91.30%) | 65631(83.13%) | 195 |
|  | **SC-1** | 79971 | 77070(96.37%) | 76289 | 72162(90.24%) | 58659(73.35%) | 187 |
|  | **SC-2** | 79132 | 75838(95.84%) | 74978 | 70749(89.41%) | 55415(70.03%) | 173 |
|  | **SC-3** | 78199 | 75867(97.02%) | 74140 | 67711(86.59%) | 54631(69.86%) | 343 |
|  | **SI-1** | 78813 | 75547(95.86%) | 74971 | 72448(91.92%) | 68828(87.33%) | 186 |
|  | **SI-2** | 78899 | 76518(96.98%) | 74582 | 67049(84.98%) | 52434(66.46%) | 266 |
|  | **SI-3** | 79781 | 77543(97.19%) | 76389 | 71757(89.94%) | 61538(77.13%) | 279 |

ASV, amplicon sequence variant.

| **Gender** | **Samples** | **Raw reads (M)** | **Clean reads (M)** | **Clean Bases (G)** | **Q30 (%)** | **GC (%)** | **Total mapped**  **(Reads, %)** | **Unique mapped**  **(Reads, %)** | **Multiple mapped**  **(Reads, %)** |
| --- | --- | --- | --- | --- | --- | --- | --- | --- | --- |
| **Female** | **Control-1** | 71.78 | 70.46 | 10.58 | 97.45 | 48.58 | 68934763 (97.83%) | 64444202 (91.46%) | 4490561 (6.37%) |
|  | **Control-2** | 77.61 | 75.69 | 11.38 | 97.51 | 48.79 | 74214637 (98.04%) | 69075454 (91.25%) | 5139183 (6.79%) |
|  | **Control-3** | 71.41 | 69.74 | 10.48 | 97.37 | 48.70 | 68315712 (97.96%) | 63258186 (90.71%) | 5057526 (7.25%) |
|  | **SL-1** | 69.85 | 68.50 | 10.29 | 97.52 | 48.49 | 67063441 (97.91%) | 62644250 (91.46%) | 4419191 (6.45%) |
|  | **SL-2** | 80.17 | 78.55 | 11.80 | 97.41 | 48.78 | 76883326 (97.88%) | 70334822 (89.54%) | 6548504 (8.34%) |
|  | **SL-3** | 78.55 | 76.91 | 11.55 | 97.47 | 48.62 | 75179055 (97.75%) | 69261257 (90.06%) | 5917798 (7.69%) |
|  | **SC-1** | 68.36 | 66.89 | 10.05 | 97.47 | 48.52 | 65452911 (97.86%) | 61141945 (91.41%) | 4310966 (6.45%) |
|  | **SC-2** | 72.42 | 70.55 | 10.60 | 97.43 | 48.67 | 68953832 (97.74%) | 64045655 (90.78%) | 4908177 (6.96%) |
|  | **SC-3** | 75.06 | 73.58 | 11.05 | 97.10 | 48.65 | 71977593 (97.83%) | 67089043 (91.18%) | 4888550 (6.64%) |
|  | **SI-1** | 79.72 | 78.10 | 11.73 | 97.16 | 48.24 | 76216752 (97.58%) | 71025417 (90.94%) | 5191335 (6.65%) |
|  | **SI-2** | 74.76 | 72.97 | 10.97 | 97.56 | 48.80 | 71394191 (97.84%) | 67315995 (92.25%) | 4078196 (5.59%) |
|  | **SI-3** | 68.69 | 66.79 | 10.04 | 97.36 | 48.73 | 65441077 (97.97%) | 60902599 (91.18%) | 4538478 (6.79%) |
| **Male** | **Control-1** | 82.56 | 80.26 | 11.73 | 97.34 | 47.50 | 77560216 (96.63%) | 74516905 (92.84%) | 3043311 (3.79%) |
|  | **Control-2** | 79.25 | 77.74 | 11.45 | 97.41 | 47.19 | 75028883 (96.51%) | 71904910 (92.49%) | 3123973 (4.02%) |
|  | **Control-3** | 80.10 | 78.31 | 11.50 | 97.42 | 46.97 | 75321897 (96.18%) | 71849321 (91.75%) | 3472576 (4.43%) |
|  | **SL-1** | 80.84 | 78.76 | 11.53 | 97.35 | 47.05 | 76123925 (96.66%) | 73059480 (92.76%) | 3064445 (3.89%) |
|  | **SL-2** | 78.64 | 76.77 | 11.25 | 97.46 | 46.86 | 73971128 (96.36%) | 70535729 (91.88%) | 3435399 (4.48%) |
|  | **SL-3** | 74.53 | 72.62 | 10.63 | 97.33 | 47.23 | 70112457 (96.55%) | 67563833 (93.04%) | 2548624 (3.51%) |
|  | **SC-1** | 80.58 | 79.05 | 11.64 | 97.27 | 47.11 | 76405896 (96.66%) | 73644857 (93.17%) | 2761039 (3.49%) |
|  | **SC-2** | 77.49 | 75.26 | 10.98 | 97.55 | 47.02 | 72516561 (96.35%) | 69309689 (92.09%) | 3206872 (4.26%) |
|  | **SC-3** | 76.51 | 74.54 | 10.91 | 97.36 | 47.45 | 72099398 (96.73%) | 69271077 (92.93%) | 2828321 (3.79%) |
|  | **SI-1** | 82.13 | 80.19 | 11.76 | 97.42 | 47.05 | 77350778 (96.46%) | 73970854 (92.24%) | 3379924 (4.21%) |
|  | **SI-2** | 80.38 | 78.75 | 11.58 | 97.59 | 47.13 | 76141591 (96.69%) | 73202719 (92.96%) | 2938872 (3.73%) |
|  | **SI-3** | 81.47 | 79.46 | 11.64 | 97.32 | 47.24 | 76901345 (96.79%) | 74170437 (93.35%) | 2730908 (3.44%) |

**Table S2 Quality assessment of transcriptome sequencing for longsnout catfish.**

M, million; G, gigabase; Q30, percentage of bases with Phred quality score ≥ 30; GC, guanine-cytosine content.
