# Supplementary material for: Combined Effects of Dietary Soybean Lecithin With Vitamin C or Inositol on Growth, Gonadal Development, and Intestinal Microbiota in Longsnout Catfish
Source: Aquac Nutr. 2026 Sep 22;2026:4061190. doi: 10.1155/anu/4061190 (PMC13595401; doi:10.1155/anu/4061190)
Supplement: Supplementary file 1 [file ANU-2026-4061190-s001.zip › Supplementary Figures-R2.docx]

**Supplementary Figures**

**Fig. S1. Electrophoretic results of sex identification of experimental fish in control group.** Sex-specific primers for longsnout catfish amplify both 992 bp and 684 bp in males and only one fragment of 992 bp in females. M: DL 2000 DNA marker; 1–10: 10 experimental fish in the first replicate bucket of the Control group.

**Fig. S2. Volcano plot of DEGs.** (A) Volcano plot of DEGs for pairwise comparisons among groups in females; (B) Volcano plot of DEGs for pairwise comparisons among groups in males; Red dots represent significantly up-regulated DEGs; blue dots represent significantly down-regulated DEGs; light pink dots represent up-regulated genes that are not statistically significant; light blue dots represent down-regulated genes that are not statistically significant; gray dots represent genes with no differential expression.

**Fig. S3.** Commonalities and differences in gene expression between male and female fish across different comparison groups. F, female; M, male.

**Fig. S4. Gene Ontology (GO) enrichment analysis of differentially expressed genes (DEGs).** (A) GO enrichment analysis for pairwise comparisons among groups in females; (B) GO enrichment analysis for pairwise comparisons among groups in males.

**Fig. S5. Kyoto Encyclopedia of Genes and Genomes (KEGG) enrichment analysis of DEGs.** (A) SL vs Control group in female; (B) SC vs Control group in female; (C) SI vs Control group in female; (D) SL vs Control group in male; (E) SC vs Control group in male; (F) SC vs SI group in male.
